# Complete Genome Sequences of Seven Uropathogenic Escherichia coli Strains Isolated from Postmenopausal Women with Recurrent Urinary Tract Infection

**DOI:** 10.1128/MRA.00700-20

**Published:** 2020-08-13

**Authors:** Belle M. Sharon, Amber Nguyen, Amanda P. Arute, Neha V. Hulyalkar, Vivian H. Nguyen, Philippe E. Zimmern, Nicole J. De Nisco

**Affiliations:** a Department of Biological Sciences, University of Texas at Dallas, Richardson, Texas, USA; b Department of Urology, University of Texas Southwestern Medical Center, Dallas, Texas, USA; University of Maryland School of Medicine

## Abstract

Uropathogenic Escherichia coli (UPEC) is the most common cause of urinary tract infection (UTI). This disease disproportionately affects women and frequently develops into recurrent UTI (rUTI) in postmenopausal women. Here, we report the complete genome sequences of seven UPEC isolates obtained from the urine of postmenopausal women with rUTI.

## ANNOUNCEMENT

Urinary tract infection (UTI) is a serious public health concern (1). The most common etiologic agent of UTI is uropathogenic Escherichia coli (UPEC) (1, 2). The lifetime incidence rate of UTI in women is >60% (3). Approximately 50% of UTIs in postmenopausal women develop into recurrent UTI (rUTI), defined as ≥2 UTIs within 6 months or ≥3 UTIs within 12 months (4–6).

UPEC strains from postmenopausal women with rUTI are understudied. The availability of complete genomes enables analysis of the mobile genetic elements and resistance genes that complicate the management of rUTI and may inform new treatment strategies. We report the complete genomes of seven UPEC strains (Table 1) isolated from the urine of consenting postmenopausal women meeting criteria for uncomplicated rUTI as part of an institutional review board (IRB)-approved study (STU 032016-006, MR 17-120) (7).

**TABLE 1:** Accession numbers, assembly parameters, and isolate characteristics of seven uropathogenic Escherichia coli isolates from postmenopausal women with rUTI

Strain	BioSample accession no.	SRA accession no.^*a*^	Total no. of reads	*N*_50_ (bp)	Read depth (×)	MLST^*b*^	GenBank accession no.	Type of contig (circular)	Total length (bp)	GC content (%)	No. of CDSs^*c*^	Plasmid replicon(s)
EcPF5	SAMN15075992	SRX8452297 (O)	270,764	8,643	273	73	CP054236	Chromosome	5,147,412	50.4	4,747	NA^*d*^
		SRX8452051 (I)	2,717,378		75		CP054237	Plasmid	131,660	52.5	147	IncFIA, IncFIB, IncFII, IncQ1
							CP054238	Plasmid	1,546	51.5	2	Col(MG828)
EcPF7	SAMN15075993	SRX8452298 (O)	261,756	3,872	122	2279	CP054232	Chromosome	4,996,527	50.7	4,685	NA
		SRX8452052 (I)	2,614,770		74		CP054233	Plasmid	129,632	51.5	158	IncFIB, IncFII, Col156
							CP054234	Plasmid	4,072	51.5	3	Unknown
							CP054235	Plasmid	1,551	51.1	1	Col(MG828)
EcPF14	SAMN15075994	SRX8452299 (O)	526,610	4,967	338	73	CP054230	Chromosome	5,129,852	50.5	4,725	NA
		SRX8452053 (I)	2,914,068		83		CP054231	Plasmid	7,154	48.6	9	Unknown
EcPF15	SAMN15075995	SRX8452300 (O)	122,027	17,034	250	394	CP054227	Chromosome	4,796,742	50.5	4,342	NA
		SRX8452054 (I)	2,373,392		70		CP054228	Plasmid	135,461	48.6	133	IncFIB, IncFII
							CP054229	Plasmid	6,431	42.6	6	Col(pHAD28)
EcPF16	SAMN15075996	SRX8452301 (O)	390,622	4,192	197	216	CP054224	Chromosome	4,721,932	51.0	4,430	NA
		SRX8452055 (I)	8,965,752		231		CP054225	Plasmid	123,935	50.5	130	IncFIA, IncFIB
							CP054226	Plasmid	5,658	43.4	7	Col(pHAD28)
EcPF18	SAMN15075997	SRX8452302 (O)	196,886	7,168	161	131	CP054219	Chromosome	5,010,549	50.7	4,679	NA
		SRX8452056 (I)	12,568,424		310		CP054220	Plasmid	128,954	50.9	146	IncFIA, IncFIB, IncFII, Col156
							CP054221	Plasmid	86,740	50.1	96	IncI1-I(Gamma)
							CP054222	Plasmid	5,167	47.5	6	Col156
							CP054223	Plasmid	1,506	50.2	2	Col(MG828)
EcPF40	SAMN15075998	SRX8452303 (O)	426,633	5,656	315	1193	CP054214	Chromosome	5,025,664	50.6	4,704	NA
		SRX8452057 (I)	2,296,172		64		CP054215	Plasmid	89,178	52.6	106	IncFIA, IncFIB, IncQ1
							CP054216	Plasmid	88,657	50.2	102	IncI(Gamma)
							CP054217	Plasmid	5,167	47.5	6	Col156
							CP054218	Plasmid	2,113	47.1	2	Col(BS512)

a The technology used is shown in parentheses. O, ONT; I, Illumina.

b MLST, multilocus sequence type.

c CDSs, coding sequences.

d NA, not applicable.

Clean-catch midstream urine was collected from seven subjects and plated onto CHROMagar Orientation (BD) and blood agar media. After overnight incubation at 37°C, well-isolated single colonies were picked for species identification by Sanger sequencing of the 16S rRNA gene and a nonredundant/nucleotide (nr/nt) database query using MegaBLAST (BLAST v2.10.0) (7, 8). Identified UPEC isolates were grown in brain heart infusion broth overnight at 37°C, and genomic DNA was extracted using a DNeasy blood and tissue kit (Qiagen).

The genomic DNA, assessed by the 260/280-nm absorbance ratio and agarose gel electrophoresis, was sequenced using Illumina and Oxford Nanopore (ONT) technologies. Illumina library preparation and sequencing were performed using the Nextera DNA Flex library prep kit and the NextSeq 500 system (300 cycles) to generate 2 × 150-bp paired-end reads. ONT libraries were prepared using the ligation sequencing kit (SQK-LSK109) and barcode expansion kit 1-12 (EXP-NBD104); then, they were sequenced on the MinION instrument using R9 FLO-MIN106 flow cells. Live fast base calling, demultiplexing, and barcode trimming were performed using ONT MinKNOW software.

Illumina reads were quality assessed and trimmed using CLC Genomics Workbench v12.0.3 trimming reads with a Phred score below 20. Reads of <15 bp were discarded. ONT reads were assessed for quality using NanoStats v1.2.0 (9) and trimmed using NanoFilt v2.6.0 (9) to retain reads with a Phred score above 7 and length greater than 200 bp (Table 1).

Hybrid assemblies of Illumina and ONT reads were completed using the Unicycler v0.4.8 pipeline (SPAdes v3.13.0, Racon v1.4.10, and Pilon v1.23) at default parameters (10–13). Assembly of all the isolates generated complete, circular chromosome and plasmid contigs. Genomes were rotated to the starting base of the *dnaA* or *repA* gene, if found. The hybrid assembly quality was assessed using QUAST v5.0.2 (14). Genome completeness was evaluated using Bandage v0.8.1 (15) and BUSCO v1 (16) with bacteria ortholog set on the gVolante server v1.2.1 (17). Coverage of core genes was 100% for all genomes. Annotation was performed using NCBI Prokaryotic Genome Annotation Pipeline v4.11 with default parameters (18, 19). The GC content and number of coding sequences were determined with Geneious Prime v2020.0.5. Multilocus sequence typing was performed using MLST v2.0 (http://www.genomicepidemiology.org/) with Escherichia coli configuration 1 (20). Plasmid replicons were identified with PlasmidFinder v2.1 (21, 22) with 90% identity and 60% minimum coverage cutoffs. Nearly all strains contained incompatibility plasmids of 88 kb to 131 kb and Col plasmids of 1.5 kb to 6.4 kb. (Table 1).

Hybrid assembly enabled the complete resolution of chromosome and plasmid sequences. These data provide insight into the plasmids harbored by diverse rUTI UPEC strains isolated from postmenopausal women.

### Data availability.

The complete sequences were deposited in GenBank under BioProject accession number PRJNA636382. The BioSample and SRA accession numbers for each isolate can be found in Table 1.

## References

[1] Flores-Mireles AL, Walker JN, Caparon M, Hultgren SJ 2015 Urinary tract infections: epidemiology, mechanisms of infection and treatment options. Nat Rev Microbiol 13:269–284. doi:10.1038/nrmicro3432. 25853778 PMC4457377

[2] Foxman B 2014 Urinary tract infection syndromes: occurrence, recurrence, bacteriology, risk factors, and disease burden. Infect Dis Clin North Am 28:1–13. doi:10.1016/j.idc.2013.09.003. 24484571

[3] Klein RD, Hultgren SJ 2020 Urinary tract infections: microbial pathogenesis, host-pathogen interactions and new treatment strategies. Nat Rev Microbiol 18:211–226. doi:10.1038/s41579-020-0324-0. 32071440 PMC7942789

[4] Dason S, Dason JT, Kapoor A 2011 Guidelines for the diagnosis and management of recurrent urinary tract infection in women. Can Urol Assoc J 5:316–322. doi:10.5489/cuaj.687. 22031610 PMC3202002

[5] Foxman B 1990 Recurring urinary tract infection: incidence and risk factors. Am J Public Health 80:331–333. doi:10.2105/ajph.80.3.331. 2305919 PMC1404686

[6] Glover M, Moreira CG, Sperandio V, Zimmern P 2014 Recurrent urinary tract infections in healthy and nonpregnant women. Urol Sci 25:1–8. doi:10.1016/j.urols.2013.11.007. 27499825 PMC4973860

[7] De Nisco NJ, Neugent M, Mull J, Chen L, Kuprasertkul A, de Souza Santos M, Palmer KL, Zimmern P, Orth K 2019 Direct detection of tissue-resident bacteria and chronic inflammation in the bladder wall of postmenopausal women with recurrent urinary tract infection. J Mol Biol 431:4368–4379. doi:10.1016/j.jmb.2019.04.008. 31002774 PMC6801050

[8] Zhang Z, Schwartz S, Wagner L, Miller W 2000 A greedy algorithm for aligning DNA sequences. J Comput Biol 7:203–214. doi:10.1089/10665270050081478. 10890397

[9] De Coster W, D'Hert S, Schultz DT, Cruts M, Van Broeckhoven C 2018 NanoPack: visualizing and processing long-read sequencing data. Bioinformatics 34:2666–2669. doi:10.1093/bioinformatics/bty149. 29547981 PMC6061794

[10] Bankevich A, Nurk S, Antipov D, Gurevich AA, Dvorkin M, Kulikov AS, Lesin VM, Nikolenko SI, Pham S, Prjibelski AD, Pyshkin AV, Sirotkin AV, Vyahhi N, Tesler G, Alekseyev MA, Pevzner PA 2012 SPAdes: a new genome assembly algorithm and its applications to single-cell sequencing. J Comput Biol 19:455–477. doi:10.1089/cmb.2012.0021. 22506599 PMC3342519

[11] Vaser R, Sovic I, Nagarajan N, Sikic M 2017 Fast and accurate de novo genome assembly from long uncorrected reads. Genome Res 27:737–746. doi:10.1101/gr.214270.116. 28100585 PMC5411768

[12] Walker BJ, Abeel T, Shea T, Priest M, Abouelliel A, Sakthikumar S, Cuomo CA, Zeng Q, Wortman J, Young SK, Earl AM 2014 Pilon: an integrated tool for comprehensive microbial variant detection and genome assembly improvement. PLoS One 9:e112963. doi:10.1371/journal.pone.0112963. 25409509 PMC4237348

[13] Wick RR, Judd LM, Gorrie CL, Holt KE 2017 Unicycler: resolving bacterial genome assemblies from short and long sequencing reads. PLoS Comput Biol 13:e1005595. doi:10.1371/journal.pcbi.1005595. 28594827 PMC5481147

[14] Gurevich A, Saveliev V, Vyahhi N, Tesler G 2013 QUAST: quality assessment tool for genome assemblies. Bioinformatics 29:1072–1075. doi:10.1093/bioinformatics/btt086. 23422339 PMC3624806

[15] Wick RR, Schultz MB, Zobel J, Holt KE 2015 Bandage: interactive visualization of de novo genome assemblies. Bioinformatics 31:3350–3352. doi:10.1093/bioinformatics/btv383. 26099265 PMC4595904

[16] Simao FA, Waterhouse RM, Ioannidis P, Kriventseva EV, Zdobnov EM 2015 BUSCO: assessing genome assembly and annotation completeness with single-copy orthologs. Bioinformatics 31:3210–3212. doi:10.1093/bioinformatics/btv351. 26059717

[17] Nishimura O, Hara Y, Kuraku S 2017 gVolante for standardizing completeness assessment of genome and transcriptome assemblies. Bioinformatics 33:3635–3637. doi:10.1093/bioinformatics/btx445. 29036533 PMC5870689

[18] Haft DH, DiCuccio M, Badretdin A, Brover V, Chetvernin V, O'Neill K, Li W, Chitsaz F, Derbyshire MK, Gonzales NR, Gwadz M, Lu F, Marchler GH, Song JS, Thanki N, Yamashita RA, Zheng C, Thibaud-Nissen F, Geer LY, Marchler-Bauer A, Pruitt KD 2018 RefSeq: an update on prokaryotic genome annotation and curation. Nucleic Acids Res 46:D851–D860. doi:10.1093/nar/gkx1068. 29112715 PMC5753331

[19] Tatusova T, DiCuccio M, Badretdin A, Chetvernin V, Nawrocki EP, Zaslavsky L, Lomsadze A, Pruitt KD, Borodovsky M, Ostell J 2016 NCBI Prokaryotic Genome Annotation Pipeline. Nucleic Acids Res 44:6614–6624. doi:10.1093/nar/gkw569. 27342282 PMC5001611

[20] Larsen MV, Cosentino S, Rasmussen S, Friis C, Hasman H, Marvig RL, Jelsbak L, Sicheritz-Ponten T, Ussery DW, Aarestrup FM, Lund O 2012 Multilocus sequence typing of total-genome-sequenced bacteria. J Clin Microbiol 50:1355–1361. doi:10.1128/JCM.06094-11. 22238442 PMC3318499

[21] Carattoli A, Hasman H 2020 PlasmidFinder and in silico pMLST: identification and typing of plasmid replicons in whole-genome sequencing (WGS). Methods Mol Biol 2075:285–294. doi:10.1007/978-1-4939-9877-7_20. 31584170

[22] Carattoli A, Zankari E, García-Fernández A, Voldby Larsen M, Lund O, Villa L, Møller Aarestrup F, Hasman H 2014 In silico detection and typing of plasmids using PlasmidFinder and plasmid multilocus sequence typing. Antimicrob Agents Chemother 58:3895–3903. doi:10.1128/AAC.02412-14. 24777092 PMC4068535

